# How Information on a Motive to Lie Influences CBCA-Based Ratings and Veracity Judgments

**DOI:** 10.3389/fpsyg.2020.02021

**Published:** 2020-08-14

**Authors:** Jonas Schemmel, Tina Steinhagen, Matthias Ziegler, Renate Volbert

**Affiliations:** ^1^Psychologische Hochschule Berlin, Berlin, Germany; ^2^Institute for Psychology, Humboldt-Universität zu Berlin, Berlin, Germany; ^3^Zentrum für Integrative Psychiatrie, Lübeck, Germany; ^4^Charité – Universitätsmedizin Berlin, Corporate member of Freie Universität Berlin, Humboldt-Universität zu Berlin, and Berlin Institute of Health, Institut für Forensische Psychiatrie, Berlin, Germany

**Keywords:** Criteria-based Content Analysis, credibility assessment, contextual information, motive to lie, truth-default theory, truth bias

## Abstract

We investigated how information on a motive to lie impacts on the perceived content quality of a statement and its subsequent veracity rating. In an online study, 300 participants rated a statement about an alleged sexual harassment on a scale based on Criteria-based Content Analysis (CBCA) and judged its veracity. In a 3 × 3 between-subjects design, we varied prior information (motive to lie, no motive to lie, and no information on a motive), and presented three different statement versions of varying content quality (high, medium, and low). In addition to anticipating main effects of both independent variables (motive information and statement version), we predicted that the impact of motive information on both ratings would be highest for medium quality statements, because their assessment is especially ambiguous (interaction effect). Contrary to our hypotheses, results showed that participants were unaffected by motive information and accurately reproduced the manipulated quality differences between statement versions in their *CBCA-based judgments*. In line with the expected interaction effect, *veracity ratings* decreased in the motive-to-lie group compared to controls, but only when the medium- and the low-quality statements were rated (truth ratings dropped from approximately 80 to 50%). Veracity ratings in both the no-motive-to-lie group and controls did not differ across statement versions (≥82% truth ratings). In sum, information on a motive to lie thus encouraged participants to consider content quality in their veracity judgments by being critical only of statements of medium and low quality. Otherwise, participants judged statements to be true irrespective of content quality.

## Introduction

In the last 30 years, a growing body of research has shown that criteria-based content analysis (CBCA; Steller and Köhnken, 1989; Volbert and Steller, 2014) validly distinguishes true from fabricated accounts (Vrij, 2008; Amado et al., 2015, 2016; Oberlader et al., 2016). Basically, this research suggests—in line with the so-called Undeutsch hypothesis (Undeutsch, 1967)—that true statements differ from fabricated accounts in their content quality, with true statements generally containing more CBCA criteria than fabricated accounts. In the field, however, the results of a content analysis based on CBCA are never considered in isolation (see Akehurst et al., 2011). Instead, when evaluating a witness’s statement, an expert in the field is inevitably confronted with additional information from, for example, studying a case file. In fact, statement validity assessment (SVA; Köhnken, 2004; Volbert and Steller, 2014), in which CBCA plays a key role, even requires the expert to actively gather more contextual information from the case file or an interview with the witness so that it can be considered in the final evaluation of a statement (Steller, 1989; Volbert and Steller, 2014). Even though this procedure aims to improve the validity of an expert’s decision making, little is known in general about the actual impact of contextual information on the content analysis of a statement using CBCA and veracity ratings (Bogaard et al., 2014). The current study aimed to shed light on this issue. Specifically, we investigated the effect of information regarding a motive to lie, because this has received particular consideration not only within the framework of SVA (Steller and Köhnken, 1989; Köhnken, 2004) but also in work on everyday deception detection (Levine et al., 2010).

### Contextual Information and CBCA: The Case of a Motive to Lie

To the authors’ best knowledge, up to now, only one study has investigated the effect of additional case information on CBCA ratings of an abuse statement. However, it did not focus on a specific type of information (Bogaard et al., 2014). Instead, in one condition, it presented information about the accused (a pertinent criminal history of the accused, child pornography found on his computer, and a reportedly hot temper). This was meant to increase the credibility of the witness (“positive information condition”). The second condition presented varying information mainly on the personality and behavior of the witness (history of lying, rebellious behavior at school, stealing money from her mother, and alleged motive for revenge). This was meant to reduce the credibility of the witness (“negative information condition”). Varying “positive” versus “negative” information had large effects of *d* = 0.83 on CBCA ratings and *d* = 1.1 on veracity ratings with “positive” information leading to higher and “negative” information to lower CBCA and veracity ratings. The authors interpreted these results as indicating a confirmation bias resulting from contextual information that can distort CBCA ratings.

These results led us to ask whether information indicating a motive to lie might have a similar impact on a content analysis based on CBCA and subsequent veracity ratings. Theoretical frameworks such as truth-default theory (TDT; Levine, 2014) assume that people usually believe in the truthfulness of a statement unless they suspect that lying has a certain benefit for the sender. Suspecting that there is a motive to lie might play a key role in deception detection, because it increases suspicion (Levine et al., 2010). Suspicion, in turn, has been shown to generally diminish truth ratings because it decreases the credibility and the perceived trustworthiness of the sender (see, e.g., Millar and Millar, 1997). As a result, a motive to lie might be interpreted as indicative of deception, whereas the absence of a motive to lie might be interpreted as indicative of honest communication. Hence, on the one hand, information that the sender has a motive to lie might decrease a witness’s perceived credibility. Information on a motive to lie might therefore result in lower ratings of content quality and lower veracity judgments. On the other hand, information suggesting that there is no motive to lie might increase the perceived credibility of the witness, thereby resulting in higher content quality ratings and veracity judgments.

However, the effect of contextual information should also depend on characteristics of the statement to be judged—that is, the content quality of the critical account—as indicated by its CBCA scores. We assume that if the statement quality itself is not sufficient for an informed decision, this creates a sense of uncertainty. Uncertainty should stimulate raters to (intentionally or unintentionally) draw on additional sources of information and heuristics to make their decision (Tversky and Kahneman, 1974; Evans, 1989). For example, in an experimental study on the analysis of polygraph charts, Elaad et al. (1994) found that the effect of prior guilt expectations concerning the examined suspect covaried with the informative value of the polygraph results. Prior guilt expectations affected their analysis results only if the indications they contained were ambiguous and not clear. Therefore, in the current study, we assumed that the influence of additional information (motive to lie) should be *relatively* small on judgments of very high- or very low-quality statements, because they should contain clear implications on content quality and veracity. However, a judgment of a medium-quality statement is less clear and rather ambiguous. Thus, additional information from the case file such as indications on a motive to lie might have a larger effect on content quality and veracity judgments, because it might facilitate the decision-making process. We consequently assumed that the influence of contextual information regarding a motive to lie would be higher when the statements to be judged are of medium content quality.

### The Current Study: General Design and Hypotheses

In the current study, we aimed to investigate how information regarding a motive to lie would influence how laypeople perceived content quality and rated the veracity of a statement. We assessed content quality using a short scale based on the CBCA taxonomy (CBCA-based rating; see below) and varied *motive information* by providing participants with information either suggesting a motive to lie or explicitly speaking against a motive to lie. We also introduced a control group that received no information regarding a motive to lie. Moreover, because we assumed that the effect of a motive to lie would depend on the content quality of the statement to be judged, we constructed *three different versions of a harassment statement with low, medium, or high content quality as indicated by their CBCA score* (see the method section for a detailed description of the study material). Based on the aforementioned arguments, we derived the following five hypotheses:

#### Hypothesis 1

We expected that providing participants with information on a motive to lie would impact negatively on CBCA-based ratings, whereas information suggesting that there was no motive to lie would have a positive impact. We thus assumed a main effect of information on a motive to lie on CBCA-based ratings, with participants receiving information on a motive to lie generally rating statement quality lower than a control group and participants with information speaking against a motive generally rating statement quality higher than a control group.

#### Hypothesis 2

We expected an interaction effect between information on a motive to lie and the statement quality on CBCA ratings. Specifically, we assumed that the expected differences between the motive groups and controls (see section “Hypothesis 1”) would be especially pronounced when judging a statement of medium content quality.

#### Hypothesis 3

We predicted a main effect of the different statement versions on veracity ratings, with veracity ratings overall reflecting the constructed quality differences between statement versions.

#### Hypothesis 4

We expected that providing participants with information on a motive to lie would impact negatively on veracity ratings, whereas information suggesting that there is no motive to lie would have a positive impact. We thus assumed a main effect of information on a motive to lie on veracity ratings, with participants receiving information on a motive to lie generally rating the statement quality higher than controls and participants with information speaking against a motive generally rating statement quality lower than controls.

#### Hypothesis 5

We expected an interaction effect between information on a motive to lie and the statement quality on veracity ratings. Specifically, we assumed that the respective differences between the motive groups and controls (see section “Hypothesis 4”) would be especially pronounced when judging a statement of medium content quality.

## Materials and Methods

### Participants

Participants were recruited via mailing lists for mainly medicine and psychology students from two universities. An e-mail providing the link to the online study invited students to participate for course credit and/or the chance to win one of three online vouchers each worth 30 Euro. The e-mail also included a short trigger warning that participants would have to read a statement depicting a sexual assault, and that this might be stressful especially to victims of sexual violence. Out of 361 participants who followed the link contained in the mail, 314 completed the whole questionnaire. Six participants with extreme processing times (less than 140 s and more than 1,800 s) on the study pages (excluding pages containing general information at the beginning and both the debriefing and prize draw after the study) were identified and excluded from the data set. Finally, another eight participants were excluded because they had indicated not speaking German on a mother-tongue level. The remaining 300 participants took an average of 386 s (*SD* = 168) to complete the study, were predominantly female (81.7%), and had an average age of 23.2 years (*SD* = 4.8 years). Virtually all of them (97%) were university students (65% psychology; others were medicine, mathematics, medical engineering, nutrition science, and molecular life science).

### Design and Procedure

We conducted an online study using the platform soscisurvey.de. Motive information (motive to lie, no motive to lie, no information) and statement version based on its content quality (low, medium, and high) were varied between participants. This resulted in a 3 × 3 between-subject study design^1^.

#### Procedure

Participants who clicked the link in the e-mail (see above) were referred to the first page of the study on soscisurvey.de and randomly assigned to one of the 3 (motive condition) ×3 (statement version) conditions. Conditions are described in more detail below.

On the first page, all participants found another, yet more detailed trigger warning along with a data protection and privacy statement according to the guidelines of the two universities from which they had been recruited. Subsequently, participants were asked for demographic information. Next, they were all told that they would be presented with a statement by Ms. K. who accused her coworker Mr. H. of having attempted to sexually assault her. Participants assigned to the “motive-to-lie” or “no-motive-to-lie” condition were then provided with more contextual information (see below). Subsequently, all participants were told that their task was to carefully read the statement on the incident provided by Ms. K. before going on to rate it according to given criteria (see Appendix 1 for a more detailed description of the criteria). After they had finished reading the statement in their assigned condition and had moved on to the rating task, participants were free to go back to the statement anytime and as often as they deemed necessary. The rating task was followed by the manipulation check (see below) before participants were thanked for taking part in the study and provided with information on how to gain a course credit and participate in the online voucher lottery in return for their participation.

#### Power Analysis

Using the software G^∗^Power (v. 3.1.9.2; Faul et al., 2007), we ran an *a priori* power analysis to calculate the sample size needed for the current study (*F* tests; ANOVA: Fixed effects, special, main effects, and interactions). As reported above, Bogaard et al. (2014) had found that more general contextual information had a large effect on CBCA ratings. However, because we were not planning a rater training but introduce a new CBCA-based measure (see below) as well as an additional independent variable (different quality versions of the statement), we anticipated a lower—that is, a medium effect size. G^∗^Power estimated that we needed a total sample size of *N* = 270 to detect a medium effect size (*f* = 0.25) with 80% power and a type-1 error probability of 5%. Because there might be multiple reasons for excluding participants especially in an online study (e.g., incomplete questionnaires, insufficient language skills, extremely long, or short processing duration), we decided to aim for an *N*∼330 in the raw data to be sure of reaching the minimum sample size to detect the predicted medium effect size.

### Study Materials^2^

#### Motive Information

Motive information was varied on three levels: a “negative” motive-to-lie condition, a “positive” no-motive-to-lie condition, and a control condition.

In the motive-to-lie condition, participants were provided with information designed to suggest a motive to lie on the part of Ms. K. Specifically, it said that Mr. H. denied all accusations. He claimed to have no private contact with her, and that the had never gone to see her in her office after work. Participants were also told that Mr. H. assumed that Ms. K. was seeking revenge because he had recently been promoted instead of her. Furthermore, it was said that another coworker had told the police that 2 days before the alleged incident, Mr. H. and Ms. K. had been arguing, and that Ms. K. had left the office of Mr. H., shouting that he would see where all this will get him.

In the no-motive-to-lie condition, participants were provided with information designed to suggest that Ms. K. had no plausible motive to lie. Specifically, participants were told that Ms. K. and Mr. H. had been working together without incident and had neither been very close nor had had any major arguments or conflicts. It was also said that Ms. K. had recently been promoted. Furthermore, according to a coworker of Ms. K. and Mr. H., a meeting 2 days before the alleged incident had gone smoothly, and there was generally a harmonious and constructive working atmosphere in the office unit.

Finally, a control condition provided no contextual information at all on a possible motive to lie. Participants were informed only that Ms. K. is accusing her coworker of an attempted sexual assault.

#### Statement Versions Based on Their Content Quality

Having read the contextual information on a possible motive to lie, participants were presented with the statement by Ms. K. We developed three different statement versions containing a low, a medium, or a high content quality as indicated by their CBCA score. Statements were varied between participants. They were developed in the following way:

First, we composed an extensive statement by Ms. K. that read as if it was transcribed word for word. She said essentially that one day, she was alone in her office after hours when Mr. H. came knocking on her office door asking to be let in. After unlocking the door to let him in, she turned to tidy up some lunch dishes from the table. This was when Mr. H. grabbed her arm, pulled her around, and tried to kiss her. Turning her head and pushing him away from her, she asked him what he was doing. When she turned around and asked him to leave, he again approached her from behind, put his arms around her, and touched her. When she finally managed to escape his arms, she opened her office door and again asked him to leave. At first, he did not, but started to try and calm her down. She then threatened to call the police and this made him leave the office. He then finally tried to explain and apologize to her, but she could not really respond to this. Instead, she closed the door in his face and locked it, which finally made him leave.

In the high-quality version, the statement was systematically enriched with various CBCA criteria generally characterizing episodic autobiographical memory such as emotions/feelings, thoughts, verbal reproductions, or perpetrator’s mental state (Steller and Köhnken, 1989). Moreover, in line with current classifications (Volbert and Steller, 2014; Volbert, 2015), we augmented the statement with CBCA criteria describing *script-deviant and/or script-irrelevant details* such as complications or unusual/peripheral details; CBCA criteria describing *memory-related shortcomings* such as spontaneous corrections, admitting lack of memory, or efforts to remember; and finally, CBCA criteria indicating a *lack of strategic self-presentation* such as self-deprecation or pardoning the perpetrator. This initial statement contained 992 words.

For the medium-quality version, CBCA criteria were deleted systematically from the initial statement. From all four categories depicted above, at least one criterion was removed so that not only the overall CBCA score but also the score within each category was lower than in the initial statement. This version contained 528 words.

Likewise, we deleted criteria systematically from the medium-quality version to again reduce the CBCA score. The resulting low-quality version finally contained 297 words. Both medium- and low-quality versions still formed coherent and self-explanatory narratives.

#### Measurements

##### CBCA-based rating

The current sample of laypersons was unfamiliar with CBCA. In many comparable studies, participants first underwent a short introduction to CBCA to prepare them for the rating task (see, e.g., Bogaard et al., 2014). We chose a different approach and developed a short rating scale based on the CBCA criteria (Steller and Köhnken, 1989; Volbert and Steller, 2014) addressing the psychological processes assumed to underlie CBCA criteria—namely, episodic memory processes, script deviation, and absence of strategic self-presentation (Volbert and Steller, 2014; Volbert, 2015). This approach allowed us to restrict the number of items and to develop CBCA wordings that laypersons would be able to comprehend because they explained the idea behind groups of criteria instead of providing many strict and abstract definitions. Overall, we constructed five CBCA-based items: “quantity of details,” “vividness,” “script deviance,” “memory-related shortcomings,” and “incrimination.” Each was rated on a 5-point scale ranging from 1 to 5 with varying pole terms. More information on scale construction and item wordings can be found in the appendix. It is important to note that the CBCA-based rating scale was designed as a measure to assess the perceived qualitative differences between the statement versions. We do not claim that it can distinguish validly between experience-based and false statements as has been shown for the original CBCA compilation, because this was not the purpose of our study.

##### Veracity rating

Participants were asked to rate whether they considered Ms. K.’s statement to be true or fabricated on a 4-point scale ranging from 1 (*fabricated*) to 4 (*true*).

##### Manipulation check

Having completed the CBCA-based rating task and the veracity rating, participants were asked to rate whether—on the basis of the contextual information provided—Ms. K. might have had a motive to falsely accuse Mr. H. on a 5-point scale ranging from −2 (*contextual information clearly suggested a motive for a false accusation*) to +2 (*contextual information spoke against a motive for a false accusation*) with a midpoint 0 (*there was no relevant contextual information*).

### Data Analysis^3^

All calculations were run with the statistical software “*RStudio*” (v. 1.1.383; RStudio Team, 2019) implemented in “*R*” (v. 3.4.1; R Core Team, 2019)^4^. Type-1 error was set at 5% for all analyses. We computed two-way ANOVAs to test our hypotheses. We used Tukey’s HSD for *post hoc* comparisons since we intended to control for type-I error inflation without losing too much test power. “Incrimination” was reversed before being used in the calculations. The score derived from the five CBCA-based items had a comparably low Cronbach’s α = 0.65, 95% CI (0.59, 0.72), across all conditions^5^. However, we measured a broad construct and intended to use the scale only for group statistics, not individual assessments. Thus, considering the low number of items and following recent recommendations for short scale constructions (Ziegler et al., 2014), we deemed the Alpha acceptable. We computed total CBCA-based rating scores based on all five items for all further calculations (see Table 1).

**TABLE 1:** Means and standard deviations of CBCA-based ratings (total score) per motive information and statement version.

Statement version	Motive manipulation			
	Motive-to-lie	No-motive-to-lie	Control group	Mean (*SD*)^1^
High quality	20.41 (2.36)	19.88 (2.64)	19.38 (3.14)	19.89 (2.74)
Medium quality	17.24 (3.04)	18.24 (2.41)	19.27 (2.59)	18.24 (2.80)
Low quality	15.29 (3.01)	16.26 (2.67)	16.56 (2.34)	16.05 (2.72)
Mean (*SD*)^2^	17.72 (3.50)	18.11 (3.96)	18.43 (3.00)	

*Numbers refer to the sum score of all five items. Standard deviations in parentheses. After alpha adjustment for multiple testing (α = 0.05, Tukey’s HSD test), post hoc tests showed no statistically significant difference between motive ininformation conditions. ^1^Refers to means and SDs across all motive manipulations. ^2^Refers to means and SDs across all statement versions.*

## Results

### Manipulation Checks

#### Motive Manipulation

We computed a two-way ANOVA^6^ with motive information and statements’ content quality as between-subject factors and the manipulation check item as the dependent variable (“Might Ms. K. have had a motive to falsely accuse Mr. H.?”). This revealed a large main effect for the motive-to-lie group, *F*(2, 291) = 72.02, *p* < 0.001, η_*p*_^2^ = 0.33, and 95% CI (0.24, 0.40). Pairwise *post hoc* Tukey’s HSD tests revealed that this effect was driven by the difference between the motive-to-lie group (*M* = -0.78, *SD* = 0.92) and both the no-motive-to-lie group [*M* = 0.53, *SD* = 0.84, *d* = 1.47, and 95% CI (1.16, 1.78)] and controls [*M* = 0.31, *SD* = 0.64, *d* = 1.37, 95% CI (1.06, 1.68), all *p*s < 0.001]. The difference between controls and the no-motive-to-lie group did not reach statistical significance despite yielding a moderate effect size [*d* = 0.29, 95% CI (0.01, 0.56), and *p* = 0.15]. Therefore, we cannot rule out the possibility that the manipulation did not work for some members of the no-motive-to-lie group. However, the missing difference between the no-motive-to-lie group and controls was presumably driven by the fact that the latter judged the contextual information as tending to dismiss a motive to lie. Because controls had received hardly any contextual information at all and were thus expected to rate the item mainly with “0” (“There was no relevant contextual information”), this finding was unexpected. Yet, it seemed to fit quite well into the theoretical framework of “truth-default theory” (TDT; see the discussion for a more detailed elaboration). We thus decided to continue with the planned analyses for all subgroups, because we deemed it interesting from an exploratory, theoretical perspective.

#### Statement Versions

A two-way ANOVA with motive information and statement version as between-subjects factors revealed a large main effect of statement version on CBCA-based ratings, *F*(2, 291) = 50.87, *p* < 0.001, η_*p*_^2^ = 0.26, and 95% CI (0.17, 0.33), with all differences between statements with high, medium, and low content quality taking the expected direction (for mean values, see Table 1). There was a large difference between high- and low-quality statements [*d* = 1.41, 95% CI (1.1, 1.72), and *p* < 0.001], a medium difference between high- and medium-quality statements [*d* = 0.60, 95% CI (0.32, 0.88), and *p* < 0.001], and a large difference between medium- and low-quality statements [*d* = 0.80, 95% CI (0.50, 1.09), and *p* < 0.01]. Hence, participants successfully differentiated the content quality of the three statement versions with the newly developed CBCA-based rating scale. This shows that the statement version manipulation was successful, and speaks for the criterion-related validity of the newly developed CBCA-based scale as a measure of content quality.

### CBCA-Based Ratings: Main Effect of Motive Information (Hypothesis 1), and Interaction Effect (Hypothesis 2)

**FIGURE 1:**
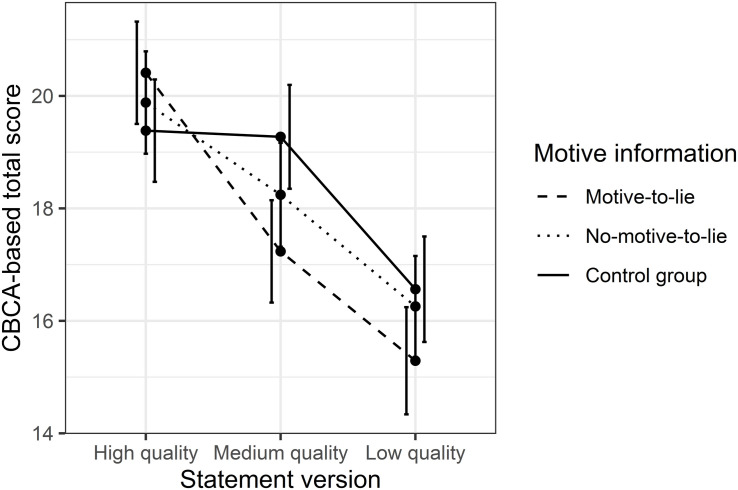
CBCA-based ratings (total scores of all five items) for the motive manipulation and the statements’ CBCA score. Error bars are 95% confidence intervals.

Contrary to our assumptions, in a two-way ANOVA with motive information and statement version as between-subjects factors, the varying information on a motive to lie had no statistically significant main effect on CBCA-based ratings, *F*(2, 291) = 1.99, *p* = 0.14, η_*p*_^2^ = 0.01, and 95% CI (0, 0.05). Nonetheless, there was a small interaction effect between both independent variables on the CBCA-based ratings, *F*(4, 291) = 2.99, *p* = 0.02; η_*p*_^2^ = 0.04, and 95% CI (0.001, 0.08). As Figure 1 shows, there was a crossover interaction effect between statements of high and medium quality in all motive groups. A closer inspection of the graph reveals that the ratings of both motive groups were lower for statements of medium than for statements of high quality. This is in line with the main effect of statement version on CBCA-based ratings (see above). On the other hand, ratings of the control group hardly differed between both statement versions. The figure suggests that this unexpected finding mainly drove the crossover interaction effect we found. Also, adjusted *post hoc* Tukey’s HSD tests revealed no significant differences of CBCA-based ratings between controls and either of the two experimental groups (0.15 ≤ *p*s ≤ 0.94; 0.17 ≤ *d*s ≤ 0.72). Hence, overall, the current data did not corroborate the expected interaction between motive information and statement quality.

### Veracity Ratings: Main Effect of Statement Version (Hypothesis 3), Main Effect of Motive Information (Hypothesis 4), and Interaction Effect (Hypothesis 5)

**FIGURE 2:**
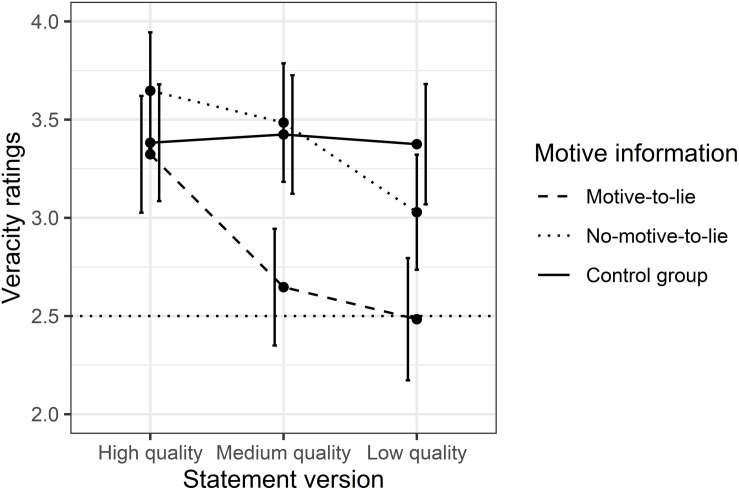
Veracity ratings (1 = fabricated to 4 = true) for the motive manipulation and the statements’ CBCA score. The dotted vertical line represents the empirical midpoint. Error bars are 95% confidence intervals.

In line with Hypotheses 3 and 4, a two-way ANOVA with motive information and statement version as between-subject factors showed a small main effect of statement version, *F*(2, 291) = 7.65, *p* < 0.001, η_*p*_^2^ = 0.05, 95% CI (0.01, 0.10), and a medium main effect of motive information, *F*(2, 291) = 13.87, *p* < 0.001; η_*p*_^2^ = 0.08, and 95% CI (0.03, 0.15). There was also a small interaction effect, *F*(4, 291) = 2.62, *p* = 0.03, η_*p*_^2^ = 0.03, and 95% CI (0, 0.07) (see Table 2 for means and standard deviations for all groups as well as Figure 2 for a graphical display).

**TABLE 2:** Means and standard deviations of veracity ratings per motive information and statement version.

Statement version	Motive manipulation			
	Motive-to-lie	No-motive-to-lie	Control group	Mean (*SD*)^1^
High quality	3.32 (0.77)	3.65 (0.60)	3.38 (0.85)	3.45 (0.75)
Medium quality	2.65* (1.10)	3.48 (0.67)	3.42 (0.87)	3.18 (0.97)
Low quality	2.48* (1.06)	3.03 (1.07)	3.38 (0.83)	2.97 (1.05)
Mean (*SD*)^2^	2.83 (1.04)	3.38 (0.84)	3.40 (0.84)	

Standard deviations in parentheses. Scale: 1 = Fabricated; 2 = Rather fabricated; 3 = Rather true; 4 = True. ^1^Means and SDs across all three motive information levels. ^2^Means and SDs across all statement versions. *Statistically significant difference to controls after alpha adjustment for multiple testing (α = 0.05, Tukey’s HSD test).

Adjusted *post hoc* Tukey’s HSD tests revealed that veracity ratings of the three motive groups showed no differences when *high-quality statements* were judged (0.85 ≤ *p*s ≤ 1.0; 0.07 ≤ *d*s ≤ 0.47). When *medium-quality* statements were judged, participants with information on a motive to lie rated statements as significantly less truthful than controls [*p* = 0.01, *d* = 0.79, and 95% CI (0.29, 1.29)], whereas there was no difference in veracity ratings between the no-motive-to-lie group and controls [*p* = 1.0, *d* = 0.08, and 95% CI (−0.40, 0.56)]. A similar pattern was revealed when *low CBCA-score statements* were rated: Whereas veracity ratings of participants with information suggesting a motive to lie were significantly lower than those of controls [*p* = 0.003, *d* = 0.93, and 95% CI (0.41, 1.45)] and approached the midpoint of the scale, there was no difference between controls and the no-motive-to-lie group [*p* = 0.80, *d* = 0.36, and 95% CI (−0.12, 0.85)]. Notably, it was particularly the veracity ratings of controls that hardly changed at all across all statement versions and remained in the upper part (i.e., the truth range) of the scale (3.38 ≤ *M*s ≤ 3.42, all *p*s∼1.0, 0.01 ≤ *d*s ≤ 0.06).

Thus, we found the predicted main effect of motive information on veracity ratings, but could not interpret it due to the type of interaction. As predicted, motive information decreased veracity ratings, but only when judging medium- and low-quality versions of the statement. We did not find the corresponding effect for the no-motive-to-lie group. In conclusion, our hypotheses were confirmed only for the motive-to-lie group.

Table 3 illustrates the veracity ratings split into truth and lie judgments (3 = Rather true and 4 = True were summarized to true judgments, 1 = Fabricated and 2 = Rather fabricated were summarized to lie judgments). Whereas controls rated statements as true in more than 80% of cases, the ratings by participants in the motive-to-lie group dropped from 88% truth judgments when judging high CBCA score statements to just above 50% truth judgments when judging medium and low CBCA score statements.

**TABLE 3:** Absolute frequencies of dichotomized veracity ratings per motive information and statement version.

Motive information	Statement version	Truth	Lie	Truth rate
Motive-to-lie	High quality	30	4	88%
	Medium quality	20	14	59%
	Low quality	16	15	52%
No- motive-to-lie	High quality	32	2	94%
	Medium quality	30	3	91%
	Low quality	26	9	74%
Control group	High quality	28	6	82%
	Medium quality	29	4	88%
	Low quality	27	5	84%

*Truth and lie judgments were originally made on a 4-point rating scale with 1 = Fabricated; 2 = Rather fabricated; 3 = Rather true; 4 = True. Ratings of 1 and 2 were summarized to “lie”; ratings of 3 and 4 were summarized to “truth.”*

### Did Participants Include Their CBCA-Based Ratings in Their Veracity Judgments?

Finally, we checked whether participants had followed the logic of CBCA and based their veracity judgments on their previous CBCA ratings. We included CBCA-based ratings as a covariate in an ANCOVA with motive information and statement version as between-subject factors and veracity ratings as the dependent variable. We assumed that by introducing CBCA-based ratings into the model, the effect of statement quality on veracity judgments (see above) would no longer be statistically significant. This would suggest a mediation effect of CBCA-based ratings on veracity judgments (Baron and Kenny, 1986). Indeed, we found no statistically significant effect of statement quality, *F*(1, 290) = 0.55, *p* = 0.58; η_*p*_^2^ = 0.001, and 95% CI (0, 0.02), whereas the model’s covariate CBCA-based ratings had a large effect, *F*(1, 296) = 83.13, *p* < 0.01; η_*p*_^2^ = 0.22, and 95% CI (0.14, 0.29). We concluded that participants generally included their CBCA-based ratings in their veracity judgments.

## Discussion

The present study investigated how information on a possible motive to lie affects judgments of witness statements. The current data did not support our assumption that information on a motive to lie would influence the perceived content quality as measured by *CBCA-based ratings*; we found only a very small main effect that was not statistically significant. Instead, CBCA-based ratings were influenced largely by the constructed content quality of the statement itself, with participants differentiating the three statement versions according to their content characteristics. Thus, participants across all conditions correctly identified the varying content quality of the statements.

However, compared to controls, we found the predicted decrease in *veracity ratings* in participants who were given information suggesting a motive to lie. Notably, this decrease was found when rating statements not only of medium but also of low content quality: The relative frequency of truth ratings decreased from 88% to just above 50%, which can be regarded as a meaningful effect. In contrast, we did not find the assumed effect for participants provided with information suggesting that there is no motive to lie: Their veracity ratings generally did not differ from those of controls. These results correspond with those of the manipulation check: Here, participants in the control and the no-motive-to-lie groups also did not differ in whether they said that the witness had a motive to lie. However, there was a large difference to the motive-to-lie group who reported having assumed a motive to lie. Thus, even though participants from all conditions correctly identified the differences between the statements regarding content quality, this content quality apparently influenced only the veracity ratings of those with information on a motive to lie: They judged statements of medium and low quality less often as true than statements of high quality.

In general, our findings contradicted those of Bogaard et al. (2014). They reported that both “positive” and “negative” information affected CBCA and veracity ratings, whereas in the current study, there was only an effect on veracity ratings. Furthermore, this was only the case for “negative” information—that is, information suggesting a motive to lie. However, Bogaard et al. (2014) did not vary statement versions systematically, presented various pieces of contextual information with rather general implications, and aggregated ratings on different types of offenses. In the current study, we investigated the effect of different statement versions, presented case-specific information on a motive to lie, and focused on sexual harassment at work. This makes the two studies difficult to compare. Moreover, Bogaard et al. (2014) did not include a control group as a baseline. Hence, strictly speaking, their data cannot distinguish between the effects of their two information conditions. Thus, the effect they reported might be driven by either “positive” information, “negative” information (as in the present study), or both. Hence, the results of both studies—although different at first glance—might actually be compatible.

### The CBCA-Based Short Scale as a Measure for Perceived Content Quality

In the current study, participants rated the content quality of the three statement versions on a CBCA-based short scale. Even though they had not undergone any specific introduction or training, their overall content quality ratings reflected the constructed quality differences between the statement versions. Moreover, participants included their CBCA-based ratings in their veracity judgments (but see limitations section). However, as already pointed out, the CBCA-based scale was constructed in order to measure the perceived content quality based on group statistics. This study did not intend to test whether it distinguishes validly between experience-based and fabricated statements as the original CBCA scale has been shown to do. Nonetheless, the current results do underline its potential as a comprehensible and short instrument for CBCA research with laypersons that does not require extensive rating training. More studies need to vary statements and their criteria-related content systematically in order to develop a reliable and valid short version of the CBCA. For example, word count could not be ruled out as a possible confounder for content quality in the current sample. Given sufficient validity of a CBCA-based short scale, however, it might one day be a handy research instrument to use when either extensive rater training is not possible or expert raters are not available.

### The Present Veracity Rating Results Are Compatible With Truth-Default Theory

Overall, it was especially the lack of differences between the no-motive-to-lie group and controls that were not in line with our hypotheses. However, a closer examination of the literature reveals that the whole pattern of results fits in well with TDT (Levine, 2014). TDT assumes that most human communication is honest (“truth bias,” see also Burgoon et al., 1995). Thus, as a default, people should (correctly) presume that others tell the truth until they have good reason to think otherwise (Levine, 2014). Hence, TDT generally predicts a strong truth bias when judging statements on sexual harassment, and that this should decrease only when a motive to lie is perceived (projected motive model; see also Levine et al., 2010). This is exactly what we observed in the current study: Most participants with no further contextual information judged all three statements to be truthful. Based on TDT and other theoretical work (Burgoon et al., 1995), it can be assumed that they did so because they had no reason to be suspicious (see also Millar and Millar, 1997). Providing information suggesting a motive to lie, however, probably induced suspicion. Hence, it had the predicted effect of generally decreasing veracity judgments. Nonetheless, this effect occurred only when the statement quality was not too high. If statement quality was high, veracity ratings were also high, even when provided with information on a motive to lie. In line with TDT, this suggests that information on a motive to lie led participants to process the statements and their characteristics more carefully (Levine et al., 2010; see also Millar and Millar, 1997, 1998).

From a TDT point of view, it is also no surprise that we did not find the predicted difference in veracity ratings between controls and the no-motive-to-lie group. This was probably because the no-motive-to-lie group was provided only with information suggesting that there was simply no reason to assume a motive to lie. As already explained above, TDT expects this to be the general default anyway. Thus, according to TDT, it was not very likely that the no-motive-to-lie manipulation as conceptualized here would induce differences to the control group in the first place. Future studies might therefore include stronger manipulations for a no-motive-to-lie group. For example, Levine et al. (2010) used confessions to committing a crime (in which a motive to lie seems extremely unlikely) that are probably better suited to elicit differences between a no-motive-to-lie and control conditions.

To conclude, the current pattern of results is quite compatible with the theoretical framework of TDT. This is especially the case for the lack of differences between the no-motive-to-lie group and the control group. However, because our considerations regarding the latter findings are *post hoc* considerations, replication studies need to test them as explicit hypotheses.

### Dual Process Theories of Social Judgment – Did Information on a Motive to Lie Induce Systematic Rather Than Heuristic Processing?

Dual-process theories have suggested two modes of information processing in social judgment tasks: one that involves attempts to thoroughly understand the available information (systematic processing) and another that involves focusing on salient and easily comprehendible cues activating well-learned judgmental shortcuts (heuristic processing; for an overview see Chaiken and Ledgerwood, 2012). Reinhard and Sporer (2008, 2010) showed that dual-process theories can also be used to understand credibility judgments. In the current study, information on a motive to lie caused participants to include verbal content (ratings) of the statements in their veracity judgments. This was neither the case in the control group nor the no-motive-to-lie group. In these conditions, participants appeared to base their decisions merely on motive information, that is, heuristic processing. Information on a motive to lie may therefore have induced systematic processing of the verbal content information. However, work on dual-process theories has stressed that systematic processing requires motivational and cognitive involvement. If this involvement is missing, heuristic processing is preferred (Reinhard and Sporer, 2010). Nonetheless, cognitive and/or motivational involvement was not manipulated in this study and yet differences in information processing between the motive groups were found. Possibly, information on a motive to lie and the incriminating information contained in the statement may have been perceived as incongruent. Information incongruence has been shown to result in primarily systematic information processing (Chaiken and Maheswaran, 1994). However, when a verbal message is ambiguous, systematic processing of its content appears to be influenced or biased by heuristic processing as well. Thus, both systematic and heuristic processing play a role when ambiguous messages are processed. This is in line with our finding that the effect of a motive to lie was greatest when statements of medium, that is, ambiguous quality were judged. To conclude, dual-process theories on social judgments provide a fruitful theoretical approach in order to understand the processes which drive the effects of contextual information on credibility judgments. More research is required to disentangle possible effects of cognitive/motivational involvement and information incongruence on information processing, when information on a motive to lie is provided.

### Information on a Motive to Lie Shifted the Focus to Content Quality: A Distortion or an Improvement of Judgment?

Given that experts using CBCA in the field are always confronted with contextual information, the question arises whether this might have negative or positive effects on CBCA and credibility evaluations. As already pointed out above, our results suggest that information on a motive to lie might influence judgments by changing how raters come to a decision. Motive information apparently made them include content quality in their veracity ratings; when no lie suspicion was induced by motive information, participants’ ratings indicated a truth bias across the different statement versions. In other words, participants who were not suspicious ignored the different statement characteristics, whereas participants confronted with information suggesting that there is a motive to lie focused on content quality. This rather speaks against a distortive effect of motive information on veracity judgment. After all, given that true statements have been shown to generally contain more CBCA criteria than fabricated statements (see, e.g., Oberlader et al., 2016), it appears to be the preferable strategy to take the statement quality into account in the judgment rather than to leave it aside. In addition, according to psychological theories, people do indeed usually lie for a reason—that is, when they have a motive to lie. Apart from TDT (see above), activation-decision-construction-action theory (ADCAT, Walczyk et al., 2014)—a comprehensive theoretical framework on the whole process of lying—assumes that people will lie only when it is necessary in order to attain personal goals. Therefore, the current results raise the question whether information on a motive to lie might even improve statement judgments, because it raises reasonable doubts and moves possibly relevant information into the focus of attention. However, whether a motive to lie actually affects judgment accuracy is an open empirical question that will require more studies that vary the veracity status of the statements to be judged. It would also be helpful to learn more about the prevalence of a motive to lie in both true and false allegations in the field.

### Limitations

Some limitations should be noted: To begin with, as already elaborated above, the no-motive-to-lie manipulation was probably not strong enough to elicit judgment differences compared to controls. Also, we did not use the original CBCA taxonomy to assess content quality, and our sample consisted of university students largely unfamiliar with credibility assessment tools. Thus, the current study is rather a study of how laypeople do credibility assessment in everyday life. Referring to dual-process theories depicted above, experts on credibility assessment might rely more on systematic processing of verbal information instead of heuristic processing. The effects of information on a motive to lie might therefore be different in a sample of experts. Furthermore, recent works have pointed out that in order to achieve sufficient power (∼80%) to detect attenuated interactions, larger sample sizes are usually needed that exceed the current one (Blake and Gangestad, 2020). Future studies might therefore collect larger sample sizes to reduce possible type-II errors and obtain more robust effects. In addition, the relatively low alpha of the CBCA-based short scale has the implication that the effects found might be lower bound estimates of the actual effects, attenuated by relatively low reliability. However, there is empirical work which suggests that test-retest correlations are much more important for a test score’s criterion validity than internal consistency estimates (McCrae et al., 2011). Consequently, future studies should also explore the stability of scores from the here proposed short scale. Finally, this study cannot state whether information on a motive to lie does indeed improve or impair judgments in the field. This question will require more studies directly assessing the impact of information on a motive to lie on judgment accuracy.

## Conclusion

Our findings imply that people overcome the typically found truth bias when they are provided with information about a motive to lie. In this situation, they take content characteristics of the relevant statement into account when judging its veracity. Thus, information on a motive to lie might have a positive effect on decision making. Future research should test the effect of information on a motive to lie in a sample consisting of CBCA experts, use the original CBCA taxonomy, and investigate the impact of varying motive information on judgment accuracy in the field.

## Data Availability Statement

All study materials, the dataset as well as the R code used for the analysis of the data can be found under the following link: https://osf.io/h564u/.

## Ethics Statement

This study was carried out in accordance with the Ethical Guidelines of the German Psychological Society (DGPs). Trigger warnings were presented to all subjects who then gave informed consent in accordance with these guidelines.

## Author Contributions

JS and RV designed the study. JS and TS developed the study materials and planned the experiment. TS programmed the online survey and collected the data. JS and TS analyzed the data. JS wrote the manuscript. MZ contributed to the statistical analyses. RV supervised the whole project. All authors discussed the results and commented on the manuscript.

## Conflict of Interest

The authors declare that the research was conducted in the absence of any commercial or financial relationships that could be construed as a potential conflict of interest.

## Footnotes

1 All data and materials not contained in the manuscript are available at osf.io/xxxx.

2 All study materials both in German and in English can be retrieved online at https://osf.io/h564u.

3 The data and the R code used to analyze the data can be found under the following link: https://osf.io/h564u.

4 Data were imported using the package “haven” (Wickham and Miller, 2019). “plyr” (Wickham, 2011) was used to name, define, and recode variables. “car” (Fox and Weisberg, 2019) was used for all ANOVA calculations, “DescTools” (Signorell, 2019) was used to calculate *post hoc* tests and effect sizes, and “MBESS” (Kelley, 2019) to calculate confidence intervals around effect sizes. All figures were created with “ggplot2” (Wickham, 2016).

5 Internal consistency of the CBCA-based ratings was computed using the R package “psych” (Revelle, 2018).

6 We did not test for either normality or equality of variances in all ANOVAs, because given appropriate sample size, results of ANOVAs are considered to be robust against violations of these assumptions (Box, 1953; Ito, 1980; Schmider et al., 2010; Mircioiu and Atkinson, 2017).

## Appendix 1

### Construction of the CBCA-Based Rating Scale

We constructed five CBCA-based items. First, with “quantity of details” and “vividness of event,” we included two criteria referring to basic *episodic memory characteristics* associated with the truthfulness of an account (Volbert and Steller, 2014; Volbert, 2015) that should be comprehensible for laypersons. Quantity of details was transformed into the item “How detailed would you rate this statement? That is, are persons, things, places, events, and actions depicted *in detail*?” (originally in German). Participants rated this item on a 5-point scale ranging from 1 (*not detailed at all*) to 5 (*very detailed*). “Vividness of the event” was transformed into the item “Does the statement draw a vivid and illustrative picture of the events?” (originally in German). Again, participants were asked for ratings on a 5-point scale ranging from 1 (*not vivid at all*) to 5 (*very vivid*).

Second, we included CBCA criteria generally based on *script-deviant information* such as unexpected complications, unusual details, or irrelevant details (Volbert and Steller, 2014; Volbert, 2015). The underlying idea is that liars rely on general script information when constructing a lie (Steller and Köhnken, 1989). This is why details deviating from general script information are assumed to occur in true accounts while being less likely in fabricated statements. The corresponding item had to depict the main idea behind script-deviant details; but, at the same time, it had to be understandable for laypersons. Hence, we asked participants “Does the description appear to you to be schematic and limited to information directly related to the allegation, or does the description contain rather plastic details that go beyond a mere description of the allegation?” (originally German). Participants were again asked for ratings on a 5-point scale ranging from 1 (*very schematic, limited to the offense*) to 5 (*very plastic, beyond the offense*).

Third, we included criteria based on indications of a *lack of strategic self-presentation* that are supposed to occur when telling the truth but not when lying (Steller and Köhnken, 1989; Volbert and Steller, 2014). Recently, lack of strategic self-presentation has been subdivided into *memory-related shortcomings* and *other problematic content/motivation-related content* (Volbert, 2015). Memory-related shortcomings include criteria such as spontaneous corrections, efforts to remember, or admitting lack of memory. These are criteria that liars usually try to avoid in order to appear confident and convincing (Maier et al., 2018). We asked participants “To what extent does the statement contain indications of spontaneous memory retrieval (e.g., insecurities, spontaneous supplementing/corrections, or admitting lack of memory)?” (originally in German). Participants were again asked for ratings on a 5-point scale ranging from 1 (*no indications of memory retrieval at all*) to 5 (*indications of memory retrieval strongly pronounced*). *Other problematic content* mainly includes lack of incrimination, that is, pardoning the perpetrator and self-deprecation (Volbert, 2015). These criteria were formerly labeled motivation-related content by Steller and Köhnken (1989). We asked “To what extent does the statement contain indications for a particular intention of Ms. K. to incriminate Mr. H.?” (originally in German). Participants had to rate this item on a 5-point scale ranging from 1 (*there was no incrimination tendency*) to 5 (*there was a strong incrimination tendency*). Because within the context of CBCA, a low incrimination tendency is associated with a higher credibility, this item was reversed compared to the other items.
